# Supplementary material for: Patterns of Intron Gain and Loss in Fungi
Source: PLoS Biol. 2004 Nov 30;2(12):e422. doi: 10.1371/journal.pbio.0020422 (PMC532390; doi:10.1371/journal.pbio.0020422)
Supplement: Table S1 — Also available at http://genes.mit.edu/NielsenEtAl/. (4.3 MB ZIP). [file pbio.0020422.st001.zip › NielsenEtAl/html/400.html]

AN5195.1.NCU06922.1.MG08132.1.FG01398.1


```
 CLUSTAL W (1.82) Multiple Sequence Alignments - Introns Inserted


Sequence 1: NCU06922.1	138 aa
Sequence 2: MG08132.1	138 aa
Sequence 3: FG01398.1	139 aa
Sequence 4: AN5195.1	138 aa
Alignment Length: 139 aa
Number Identitical Residues: 113 aa
Alignment Score (without introns) 4753


MG08132.1 	-MSGEAWLYLFAVLVNAVNLFLQVFFTIMYSDLEC2DYINPIDLCNRLNTYIIPEAAVHG
NCU06922.1	-MSGEAWLYLFAVLINAVNLFLQVFFTIMYSDLEC2DYINPIDLCNRLNTYIIPEVAVHG
FG01398.1 	MMSGEAWLFLLSVLINAVNLFLQVFFTIMYSDLEC2DYINPIDLCNRLNTYIIPEAAVHG
AN5195.1  	-MSGEAWLYLLAVLINAVNLFLQVFFTIMYSDLEC2DYINPIDLCNRLNAYIIPEAGVHA
          	 *******:*::**:******************** *************:*****..**.

MG08132.1 	FLTFIFLINGYWLPLILNLPLLGWNIKK2IVDNAHLLDATEIFRKLNVHKK~ESFIKLGF
NCU06922.1	FLTFLFLINGYWLPLVLNLPLLAWNVKK2IVDNAHLLDATEIFRKLNVHKK0ESFTKLGF
FG01398.1 	FLTFLFLINGYWVPLILNLPLLGWNVKK2IVDNTHLLDATEIFRKLNVHKK0ESFFKLGF
AN5195.1  	FLTFLFVINGYWLAIALNLPLLAFNAKK2IYDNQHLLDATEIFRKLNVHKK0ESFIKLGF
          	****:*:*****:.: ******.:* ** * ** ***************** *** ****

MG08132.1 	HLIMFFFYLYSMIVALIKDESH
NCU06922.1	HLILFFFYLYSMIVALIRDEAH
FG01398.1 	HLIMFFFYLYSMIVALIRDESS
AN5195.1  	HLLMFFFYLYSMIVALIRDESH
          	**::*************:**:
```
